# Development of head-to-head and longitudinal CycleGAN algorithm for MRI harmonization: validation in follow-up MRI evaluation in patients with brain metastasis

**DOI:** 10.1038/s41598-026-43755-7

**Published:** 2026-03-11

**Authors:** Hosung Hwang, Hyeon-Ung Choi, Hyunjae Jeong, Hyun-Woo Lim, Sang Won Jo, Young Hun Jeon, Seung Hong Choi, Roh-Eul Yoo, Joon Kyung Seong

**Affiliations:** 1 Department of Radiology, Seoul National University College of Medicine, Seoul, Republic of Korea; 2 Department of Pharmaceutics, Center for Pharmacometrics and Systems Pharmacology, College of Pharmacy, University of Florida, Orlando, FL USA; 3 Department of Artificial Intelligence, Korea University, Seoul, South Korea; 4 Department of Radiology, Dongtan Sacred Heart Hospital, Hallym University College of Medicine, Hwaseong-si, Gyeonggi-do, Republic of Korea; 5 Department of Radiology, Seoul National University Hospital, Seoul, Republic of Korea; 6 School of Chemical and Biological Engineering, Seoul National University, Seoul, Republic of Korea; 7 Center for Nanoparticle Research, Institute for Basic Science (IBS), Seoul, Republic of Korea; 8 School of Biomedical Engineering, Korea University, Seoul, South Korea; 9 Department of Radiology, Seoul National University Hospital, Seoul National University College of Medicine, 101, Daehangno, Jongno-gu, Seoul, 03080 Republic of Korea; 10 Department of Artificial Intelligence, School of Biomedical Engineering, Korea University, 145 Anam-ro, Seongbuk-gu, Seoul, South Korea

**Keywords:** Brain metastasis, Follow-up, Harmonization, Head-to-head and longitudinal CycleGAN, Interscanner variability, MRI, Cancer, Medical research, Neuroscience, Oncology

## Abstract

Various harmonization methods have been employed for obtaining MRI from different scanners. However, no study has yet focused on the clinical utility of the CycleGAN technique in reducing MRI interscanner variability for patients with brain metastasis across longitudinal visits. We developed a head-to-head and longitudinal CycleGAN-based deep learning (DL) algorithm for MRI harmonization and validated its utility for follow-up (FU) MRI evaluation in patients with unchanged brain metastasis, who had FU MRI taken using a different MRI scanner. We trained the head-to-head and longitudinal CycleGAN to generate harmonized second postcontrast 3D T1W MR images with similar image impressions as the initial postcontrast 3D T1W MR images. The image similarity scores between the baseline (BL) and harmonized FU images were higher than those between the baseline and original FU images. As compared with baseline, differences in the CNRs of brain subregions were lower for the harmonized FU images than for the original FU images. More cases were read to be unchanged on the harmonized FU images than on the original FU images in terms of border, size, and contrast enhancement at a higher level of diagnostic confidence. The proposed CycleGAN algorithm may potentially decrease false positivity for the diagnosis of progression in FU MRI evaluation of brain metastasis.

**Supplementary Information:**

The online version contains supplementary material available at 10.1038/s41598-026-43755-7.

## Introduction

Intracranial metastasis is a major cause of morbidity and mortality among cancer patients. The incidence of intracranial metastasis among cancer patients is 5 per 1000 person years and most cases develop from primary lung cancer. Patients with intracranial metastasis have shorter survival than those without, with an average survival time of approximately 30.9 months^1^.

The management of patients with brain metastasis is important and sometimes demanding, and several factors, such as tumor histology, primary disease status, number of brain lesions, lesion size, and patients’ performance status, may influence the decision-making process^2^. In particular, correctly assessing the change in size of contrast-enhancing lesions on follow-up (FU) MRI is crucial for accurate and timely patient management. However, the diversity of MRI techniques makes it difficult to standardize acquisitions from different scanners, which yields contrast variations in the resultant MRI images^3^, even among images that actually have identical tumor distributions. In some institutions, different types of MRI machines are installed, introducing interscanner differences even in a single patient’s FU database. Unstandardized acquisition not only hinders clinical assessment but also potentially impedes research in more general settings, such as clinical trials and multicenter studies.

MRI harmonization can be a fundamental technique for improving reproducibility in multivendor or multicenter studies. Harmonization methods have been proposed to remove undesirable scanner effects or address the incomparability among MRI images, thus improving the statistical power and generalizability^4^.

Numerous harmonization methods have been developed to address scanner-related variability in MRI data. Traditional approaches often involve intensity normalization techniques such as histogram matching at the image level, or statistical frameworks such as ComBAT, which was originally designed to correct batch effects in feature-level measurements by standardizing distributional characteristics across batches^5^. These methods typically aim to standardize image intensities or reduce unwanted technical variability while preserving biologically relevant information.

However, with the rapid advancement of machine learning, especially deep learning, new methodologies have emerged that leverage neural networks to achieve more sophisticated and robust harmonization^6^. In terms of model architecture, the approaches can be classified into U-Nets, GANs, VAEs, flow-based generative models, and transformers.

CycleGAN, a type of GAN designed for unpaired image-to-image translation, has demonstrated promising results in this domain^7^. This model utilizes a cycle-consistency loss, which ensures that transformations between the source and target domains are reversible, thereby preserving structural information and supporting training without paired data. There have been attempts to utilize CycleGAN for image-to-image translation between different imaging modalities or different MRI sequences^8–10^. Recently, K. Gebre et al. implemented CycleGAN techniques on T1-weighted (T1W) MRI images to stylize GE into Siemens, and achieved the highest performance in terms of intraclass correlations in a study comparing different harmonization methods^11^. However, no study has yet focused on the clinical utility of the CycleGAN technique in reducing MRI interscanner variability for patients with brain metastasis across longitudinal visits. Therefore, the purpose of our study was to develop a paired CycleGAN-based deep learning algorithm for head-to-head postcontrast 3D T1W MR image harmonization and to validate its utility for FU MRI evaluation in patients with brain metastasis.

## Results

### Baseline characteristics of study participants

The baseline characteristics of the final study population are summarized in Table 1. The cohort consisted of patients with brain metastases, with balanced sex distribution and a median FU interval of approximately three months.

**Table 1:** Baseline clinical characteristics.

	Patients (*n* = 88)
Age (years)*	66 (61–72)
Sex (male: female)	44:44
Time interval between baseline and follow-up imaging (months)*	3.0 (2.7−6.1)
Primary tumor	Lung cancer (71) Breast cancer (5) Renal cell carcinoma (2) Others (10)**
Target lesion size at baseline (mm)^†^	10.6 ± 9.5

*Note* Unless otherwise indicated, data represent numbers of patients.

^*^ Data are reported as medians (IQRs).

^**^Others include tongue cancer, bone adenocarcinoma, pancreatic neuroendocrine tumor, advanced gastric cancer, leiomyoma, nasopharyngeal carcinoma, papillary thyroid cancer, intrahepatic cholangiocarcinoma, and unknown primary tumor.

^†^ Data are means ± standard deviations.

### Results of ablation study

Applying the original matching loss improved the performance compared to the setting where no original matching loss was applied (${\lambda}_{oml}=0)$ (Table 2). Moreover, as the ${\lambda}_{oml}$ increased, a general improvement was observed across the similarity metrics under both conditions: Baseline vs. Harmonized FU (FU→BL) and Baseline vs. Reconstructed BL (BL→FU→BL). Notably, ${\lambda}_{oml}=10$ achieved the best balance across both FU→BL (0.866 ± 0.081 [SSIM]; 26.21 ± 5.67 [PSNR]; 0.056 ± 0.036 [LPIPS]; *P* <. 001, respectively) and BL→FU→BL (0.970 ± 0.030 [SSIM]; 33.66 ± 4.40 [PSNR]; 0.026 ± 0.018 [LPIPS]; *P* <. 001, respectively) images. The results indicate that increasing the coefficient of original matching loss enhances the structural preservation of the images. Based on these findings, we selected ${\lambda}_{oml}=10$ as the optimal hyperparameter for our model.

**Table 2:** Ablation study of original matching loss (${{\uplambda}}_{\mathrm{o}\mathrm{m}\mathrm{l}}$).

Std Method	Mean					
	BL vs. Harmonized FU (BL vs. FU→BL)			BL vs. Reconstructed BL (BL vs. BL→FU→BL)		
	SSIM	PSNR	LPIPS	SSIM	PSNR	LPIPS
${\lambda}_{oml}$ = 0	0.842 ± 0.086	25.02 ± 5.49	0.068 ± 0.042	0.951 ± 0.040	31.30 ± 4.19	0.026 ± 0.021
${\lambda}_{oml}$ = 1	0.852 ± 0.082***	25.35 ± 4.99***	0.065 ± 0.041***	0.948 ± 0.037***	30.10 ± 4.12***	0.033 ± 0.024***
${\lambda}_{oml}$ = 5	0.862 ± 0.079***	25.72 ± 5.39***	0.061 ± 0.041***	0.966 ± 0.023***	32.39 ± 4.51***	0.025 ± 0.014***
${\lambda}_{oml}$ = 10	0.866 ± 0.081***	26.21 ± 5.67***	0.059 ± 0.036***	0.970±0.030***	33.66 ± 4.40***	0.026 ± 0.018***
${\lambda}_{oml}$ = 15	0.863 ± 0.080***	26.26 ± 5.78***	0.059 ± 0.036***	0.966 ± 0.036***	32.38 ± 5.01***	0.026 ± 0.021**

Note. Data are means ± standard deviations. The similarity metrics were compared slice-wise for varying coefficients of original matching loss under two conditions. The best results are indicated in bold. Statistical comparisons were performed using the Wilcoxon signed-rank test for paired samples. *P* values indicate comparisons with the original CycleGAN without Identity Loss (${\lambda}_{oml}$ = 0). *, **, and *** denote *P* < .05, *P* < .01, and *P* < .001, respectively.

BL = baseline; BL2FU2BL = baseline to follow-up to baseline; FU = follow-up; FU2BL = follow-up to baseline; LPIPS = learned perceptual image patch similarity; OML = original matching loss; PSNR = peak-signal-to-noise ratio; SSIM = structural similarity index measure.

### Comparison with other harmonization methods in unseen test set A

Table 3 shows the comparison between the proposed network and the existing harmonization methods for unseen test patients. For histogram matching, Pix2Pix, and all CycleGAN-based variants, the PSNR scores between baseline and harmonized FU images were significantly higher than those between baseline and original FU images (*P* < .01 for Pix2Pix and original CycleGAN with identity loss; *P* = .001 for original CycleGAN without identity loss; *P* < .001 for others).

**Table 3:** PSNR and SSIM scores between baseline and FU images in unseen test set A.

	PSNR (vs. BL)	*P* Value	SSIM (vs. BL)	*P* Value
Original FU	20.37 ± 1.84	-	0.818 ± 0.037	-
Histogram Matching	22.38 ± 2.31	< 0.001	0.842 ± 0.031	< 0.001
Pix2Pix	22.04 ± 2.19	< 0.01	0.823 ± 0.053	0.409
STGAN	**22.84 ± 2.68**	< 0.001	0.820 ± 0.050	0.561
Original CycleGAN (with Identity Loss)	22.31 ± 2.45	< 0.01	0.844 ± 0.031	0.083
Original CycleGAN (without Identity Loss)	22.37 ± 2.56	0.001	0.858 ± 0.034	< 0.001
CycleGAN (Ours)	22.61 ± 2.70	< 0.001	**0.860 ± 0.043**	< 0.001

Note. Data are means ± standard deviations. CycleGAN (Ours) incorporates an additional original matching loss with a coefficient of 10 (${\lambda}_{oml}=10)$, while all other parameters are consistent with the original CycleGAN without Identity Loss. Adversarial loss (${\lambda}_{adv})$ and Cycle Consistency loss (${\lambda}_{ccl})$ are fixed at 1 and 5, respectively. Each scores was evaluated volume-wise, and LPIPS was excluded since it is originally designed for 2D image comparison and is not directly applicable to 3D volume data. For each harmonization method, similarity scores obtained from BL–harmonized FU pairs were statistically compared with those from BL–original FU pairs using the Wilcoxon signed-rank test. *P* values less than 0.05 were considered statistically significant.

BL = baseline; FU = follow-up; PSNR = peak signal-to-noise ratio; SSIM = structural similarity index measure.

STGAN showed a significant improvement in PSNR compared with the original FU images (*P* < .001); however, the improvement in SSIM did not reach statistical significance (*P* = .561). Notably, the proposed model yielded a slightly higher SSIM than the original CycleGAN without the Identity Loss (0.860 vs. 0.858), although this difference was marginal. As shown in Table 4, the CNR differences between baseline and harmonized FU images were significantly reduced by all learning-based harmonization methods compared with the original FU images across all brain regions (mostly *P* < .001). In contrast, histogram matching generally increased CNR discrepancies or failed to reduce them in most regions. Among the deep learning approaches, the proposed model demonstrated superior performance by achieving the smallest CNR differences in the majority of regions, including the Brainstem, cerebellar white matter, cerebellar gray matter, and Pallidum. These results indicate that our method outperforms not only the incomplete CycleGAN variants but also the supervised Pix2Pix and STGAN in these specific areas.

**Table 4:** Differences in the CNRs between baseline and FU images in unseen test set A.

Regions	Original FU		Histogram Matching		*P* Value	Pix2Pix		*P* value		STGAN		*P* Value		CNR Difference					
														Original CycleGAN (with Identity Loss)	*P* value	Original CycleGAN (without Identity Loss)	*P* Value	CycleGAN (Ours)	*P* Value
Amygdala	0.623 ± 0.266		0.651 ± 0.234		0.37	0.225 ± 0.194		< 0.001		0.221 ± 0.120		< 0.001		0.241 ± 0.151	< 0.001	**0.193 ± 0.105**	< 0.001	0.197 ± 0.109	< 0.001
Brainstem	0.988 ± 0.268		1.088 ± 0.304		< 0.05	0.321 ± 0.226		< 0.001		0.409 ± 0.159		< 0.001		0.282 ± 0.128	< 0.001	0.328 ± 0.223	< 0.001	**0.260 ± 0.159**	< 0.001
Caudate	0.542 ± 0.205		0.558 ± 0.175		0.49	0.159 ± 0.136		< 0.001		**0.146 ± 0.082**		< 0.001		0.228 ± 0.112	< 0.001	0.187 ± 0.109	< 0.001	0.169 ± 0.085	< 0.001
Cerebellum WM	0.995 ± 0.249		1.076 ± 0.315		0.05	0.300 ± 0.196		< 0.001		0.365 ± 0.146		< 0.001		0.260 ± 0.173	< 0.001	0.330 ± 0.201	< 0.001	**0.243 ± 0.167**	< 0.001
Cerebellum GM	0.408 ± 0.209		0.470 ± 0.181		0.05	0.180 ± 0.090		< 0.001		0.156 ± 0.104		< 0.001		0.187 ± 0.129	< 0.001	0.174 ± 0.118	< 0.001	**0.143 ± 0.087**	< 0.001
Cerebral WM	0.643 ± 0.191		0.652 ± 0.204		0.75	0.181 ± 0.209		< 0.001		0.181 ± 0.126		< 0.001		0.194 ± 0.135	< 0.001	**0.132 ± 0.106**	< 0.001	0.163 ± 0.097	< 0.001
Insula Ctx	0.324 ± 0.208		0.414 ± 0.188		< 0.05	0.164 ± 0.231		< 0.01		**0.117 ± 0.100**		< 0.001		0.203 ± 0.156	< 0.05	0.151 ± 0.137	< 0.01	0.150 ± 0.147	< 0.001
Pallidum	0.942 ± 0.223		1.005 ± 0.266		0.12	0.243 ± 0.232		< 0.001		0.237 ± 0.157		< 0.001		0.287 ± 0.120	< 0.001	0.251 ± 0.128	< 0.001	**0.234 ± 0.110**	< 0.001
Putamen	0.704 ± 0.229		0.705 ± 0.242		0.96	**0.156 ± 0.138**		< 0.001		0.245 ± 0.123		< 0.001		0.217 ± 0.133	< 0.001	0.183 ± 0.098	< 0.001	0.168 ± 0.097	< 0.001
Thalamus Proper	0.774 ± 0.240		0.882 ± 0.277		< 0.01	0.199 ± 0.204		< 0.001		0.236 ± 0.116		< 0.001		0.234 ± 0.125	< 0.001	**0.196 ± 0.147**	< 0.001	0.211 ± 0.111	< 0.001

Note. Data are means ± standard deviations. The smallest CNR difference for each region is indicated in bold. Paired t-tests were used for statistical comparison, as the variables satisfied normality assumptions, to assess statistical significance compared to the original FU images.

BL = baseline; CNR = contrast-to-noise ratio; Ctx = cortex; FU = follow-up; GM = gray matter; WM = white matter.

### Reader study for the evaluation of clinical utility of the proposed model

For the lesion border, more cases were read to be unchanged on the harmonized FU images than on the original FU images by both readers (reader 1: 68% [88/129] vs. 55% [71/129], *P* = .03; reader 2: 71% [91/129] vs. 50% [64/129], *P* < .001) (Table 5). Similarly, both readers found more cases to have unchanged lesion sizes on the harmonized FU images than on the original FU images (reader 1: 30% [38/129] vs. 18% [23/129], *P* = .049; reader 2: 44% [57/129] vs. 36% [46/129], *P* < .001). Both readers assessed more cases to be unchanged in terms of the contrast enhancement of the lesions on the harmonized FU images than on the original FU images (reader 1: 47% [61/129] vs. 35% [45/129], *P* = .04; reader 2: 56% [72/129] vs. 45% [58/129], *P* = .02). With regard to the internal morphology of the lesions, reader 2 assessed a greater number of cases as unchanged on the harmonized FU images than on the original FU images (88% [113/129] vs. 74% [96/129], *P* = .002). The diagnostic confidence was significantly higher with the harmonized FU images than with the original FU images for both readers (Reader 1: 4.3 ± 0.8 vs. 3.4 ± 0.9, *P* <.001; Reader 2: 4.1 ± 0.6 vs. 3.8 ± 0.7, *P* = .004).

**Table 5:** Reader study for lesion characterization.

	Original FU (*n* = 129)	Harmonized FU (*n* = 129)	*P* Value
Reader 1			
Border	71 (55)	88 (68)	0.03
Size	23 (18)	38 (30)	0.049
Contrast enhancement	45 (35)	61 (47)	0.04
Internal morphology	97 (75)	93 (72)	0.67
Reader 2			
Border	64 (50)	91 (71)	< 0.001
Size	46 (36)	57 (44)	0.03
Contrast enhancement	58 (45)	72 (56)	0.02
Internal morphology	96 (74)	113 (88)	0.002

Note. Unless otherwise indicated, data represent numbers of cases interpreted to be unchanged on the FU images (percentages). *P* values were calculated using the McNemar test to compare original and harmonized FU images for each reader.

**Fig. 1:**
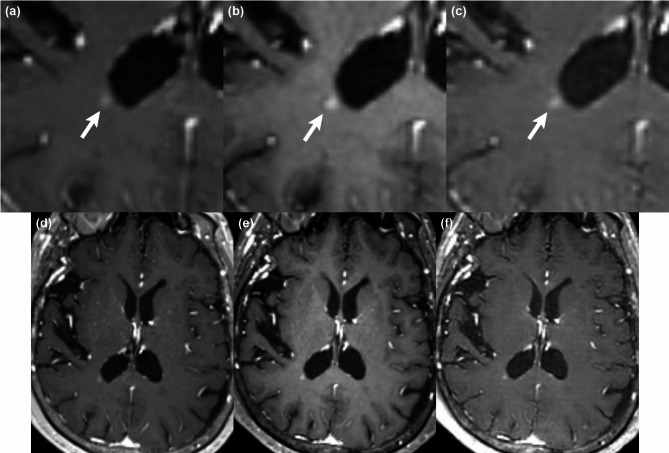
An 83-year-old woman with lung cancer. As compared with the baseline image (**a**), a small contrast-enhancing lesion (arrow) at the right temporal lobe appears slightly more discrete and larger due to a higher degree of lesion-to-white matter contrast on the original FU image (**b**). On the harmonized FU image (**c**), the lesion appears similar to that of the baseline image in terms of the border, size, and contrast enhancement. Note that the gray matter-to-white matter contrast is higher on the original FU image (**e**) than on the baseline (**d**) or harmonized FU image (**f**). The baseline and FU images were scanned with Magnetom Skyra (Siemens Healthineers) and Ingenia 3.0T CX (Philips Healthcare) MRI scanners, respectively.

**Fig. 2:**
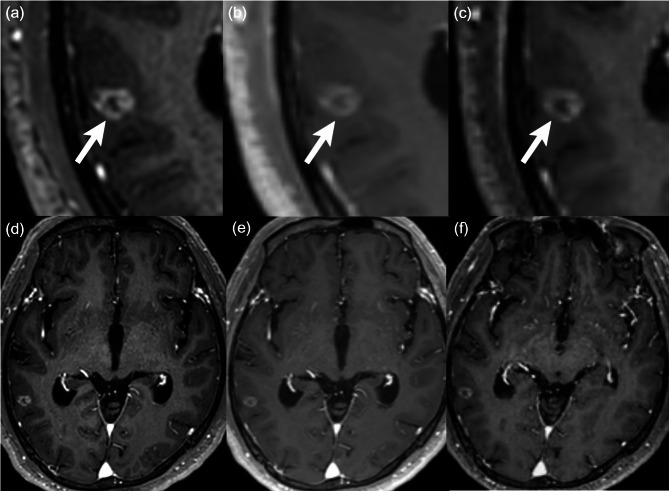
A 74-year-old man with lung cancer. As compared with the baseline image (**a**), a small contrast-enhancing lesion (arrow) at the right temporal lobe appears slightly less discrete due to a lower degree of lesion-to-gray matter contrast on the original FU image (**b**). On the harmonized FU image (**c**), the lesion appears similar to that of the baseline image in terms of the border and contrast enhancement. Note that the gray matter-to-white matter contrast is lower on the original FU image (**e**) than on the baseline (**d**) or harmonized FU image (**f**). The baseline and FU images were scanned with Ingenia 3.0T CX (Philips Healthcare) and Magnetom Skyra (Siemens Healthineers) MRI scanners, respectively.

In addition, quantitative analysis of the target lesions showed that the harmonized FU images had better alignment of lesion boundaries and greater volumetric similarity relative to baseline, as compared with the original FU images in test set A. The harmonized FU images showed lower average Hausdorff distances than the original FU images for both readers (Reader 1: 2.41 ± 1.17 vs. 2.65 ± 1.26, *P* = .02; Reader 2: 2.33 ± 1.11 vs. 2.66 ± 1.56, *P* = .02). The Dice coefficient scores were higher on the harmonized FU images than on the original FU images for both readers (Reader 1: 0.73 ± 0.17 vs. 0.67 ± 0.14, *P* = .03; Reader 2: 0.71 ± 0.16 vs. 0.66 ± 0.15, *P* = .03). Representative images of patients with brain metastases from lung cancer are shown in Figs. 1 and 2.

### Comparison with other harmonization methods and reader study in the test set B (Change Set)

Detailed results for comparison with other harmonization methods and reader study in the test set B are provided in the supplementary results and supplementary Tables 1–3. The harmonized FU images had higher PSNR and SSIM scores and reduced CNR differences, compared to the original FU, as in the test set (A) Regarding CNR differences, while our model achieved the smallest difference in cerebellar gray matter, Pix2Pix showed the lowest CNR differences across the majority of regions in Test Set (B) However, this numerical convergence in Pix2Pix was accompanied by lower PSNR scores.

In the reader study using our proposed model, the incidence of cases read to be ‘changed’ did not significantly differ between original FU images and harmonized FU images. The diagnostic confidence either increased or remained similar after the harmonization.

## Discussion

In our study, we developed and validated a paired CycleGAN-based deep learning network for the harmonization of postcontrast 3D T1W images obtained from patients with brain metastasis using different MRI scanners from different vendors. In our datasets with the baseline and FU imaging performed using different MRI scanners, our model generated the FU images that had greater similarity with the baseline images than did the original FU images, as indicated by higher image similarity performances and smaller differences in the CNRs of brain subregions. The paired CycleGAN-based deep learning algorithm increased diagnostic confidence in assessing various lesion characteristics—such as border, size, and contrast enhancement—during the FU MRI evaluation of brain metastasis patients.

The most important imaging sequence for evaluating brain metastasis is the T1W sequence following intravenous administration of a gadolinium-based contrast agent^12^. For the detection of small brain metastases, 3D magnetization-prepared (IR-prepped) gradient recalled echo (GRE) pulse sequences, including magnetization-prepared rapid acquisition with gradient echo (MPRAGE) and turbo field echo (TFE), are widely used for postcontrast T1W imaging owing to their universal availability, robustness, high signal-to-noise ratios, and superior distinction between GM and WM ^13^. Furthermore, the consensus recommendations for a standardized brain tumor imaging protocol for brain metastases emphasize that any given patient ideally needs to be scanned using the same MRI scanner platform and the same imaging protocol at all scan time points to ensure the accurate evaluation of imaging changes over time^13^. Nonetheless, the limited number of capable MRI scanners along with a large demand for FU MRI in patients with brain metastases often make it difficult to adhere to this recommendation in routine clinical practice.

Accordingly, various methods for the harmonization of medical images have been employed for obtaining MRI data from different scanners. Intensity histogram matching^14^, a conventional post-processing technique using cumulative histograms of source and target images, aims to mitigate the difference in image intensity across different scanners but may eliminate informative local variations in intensity. Therefore, statistical methods have been employed to normalize the differences in image intensity at the voxel level^15^. However, the constraints imposed by frequent adjustments when introducing new images with specific characteristics pose a considerable challenge for clinical application. This requires a specific number of subjects to be scanned at every site or with every scanner for training, which is a condition seldom met in practice.

Modern approaches to MRI harmonization employing deep learning methods have emerged as cutting-edge alternatives to address this issue^16,17^. In particular, generative models with an adversarial network have shown outstanding performance in aligning image distributions between domains. CycleGAN^18^ is a popular generative model that has been successfully applied to a wide range of image-to-image MRI translation tasks^11,19^. In a comparison study of six different methods for harmonizing the brain cortical thickness of dementia patients, CycleGAN was proved to be the best performing deep learning method^11^. In addition, Zhang et al. demonstrated that a slightly modified switchable CycleGAN outperformed the original CycleGAN model on cross-contrast MRI image synthesis in pediatrics^19^.

Traditional CycleGAN has its own strength in not requiring paired datasets. However, providing unpaired data to the model can lead to the loss of important MRI information, including structural coherence of the longitudinal FU images. Therefore, we enhanced the model by integrating the structure of the existing CycleGAN model and adding the original matching loss, which enforces voxel-wise consistency between paired baseline and FU images from the same subject, enabling direct longitudinal harmonization across scans obtained from different MRI scanners. This allowed the model to maintain the spatial information of the source data, allowing it to reflect information located at the same coordinates during the training process. By maintaining spatial information in brain regions, we could preserve the structural coherence in the longitudinal FU images. Ultimately, our model allowed us to convert MRI images to any desired scanner style while preserving the anatomical details and clinical relevance.

We evaluated the concreteness of our deep learning network in a selected group of brain metastasis patients who had stable lesions over serial FU MRIs. The observed improvements in image similarity metrics and CNR across subregions highlight the enhanced capability of our approach to preserve anatomical consistency and outperform previous methods. Specifically, our results indicate that the proposed loss term enhances longitudinal consistency and stability, even when the overall test set performance remained only marginally superior to the original CycleGAN without Identity Loss, without statistical significance. In addition, a statistically significant unification of quantitative evaluation metrics highlighted the concreteness of image translation. From a clinical perspective, more cases were read to be unchanged on the harmonized FU images in terms of the lesion border, size, and contrast enhancement by both readers, which may potentially decrease false positivity for the diagnosis of progression. The difference in image contrast between the contrast-enhancing lesions and the normal-appearing WM between the two MRI scanners may have resulted in more cases being interpreted as having changes in lesion borders and sizes on the original FU images. This finding aligns with our quantitative analysis results, which showed that the differences in the CNRs of brain regions, as compared with baseline, were lower for the harmonized FU images than for the original FU images. Notably, the increased diagnostic confidence in both readers is likely to have clinical relevance for future applications, demonstrating the efficacy of our deep learning-based harmonization algorithm in aiding diagnostic decision-making.

Moreover, in the subset of patients who had progressive or regressive changes over the FU, the harmonization algorithm also successfully accounted for image changes resulting from the subject’s disease status, supporting the generalizability of our model to datasets with varying lesion characteristics. With regard to quantitative evaluation, supervised approaches such as Pix2Pix showed smaller CNR differences in Test B, however, this likely reflects partial loss of disease-related imaging features rather than true harmonization. Because Test Set B includes disease progression, a robust model must preserve biological changes while removing scanner-induced variance. Pix2Pix’s pixel-wise L1 objective over-smoothed high-frequency signals and biased progressing lesions toward baseline intensities, artificially lowering regional variance and reducing diagnostic detail, consistent with its lower SSIM (Supplementary Table 1). Our model instead reduced background variance while maintaining longitudinal disease characteristics and achieved the highest PSNR among deep learning methods, whereas Pix2Pix showed the lowest PSNR. However, as some quantitative evaluation results indicate higher performance in cases with progressing metastases, future comparative studies between patients with and without progressing lesions are needed to further validate the model’s robust applicability across diverse clinical scenarios.

Our study had several limitations. First, this was a retrospective study based on a relatively small study population, and thus, the results could have been influenced by selection bias. Second, there were cases where multiple datasets were derived from a single patient, depending on the length of the FU period. This data clustering might have influenced the model training process. Third, we tested our model only for MRI harmonization between two MRI scanners from two different vendors (Philips Healthcare and Siemens Healthineers). To generalize our algorithm for routine clinical settings, further prospective studies including various MRI scanners from other vendors with varying T1-weighted scan protocols are warranted. Fourth, a direct reader comparison between the harmonization methods was not performed, as their quantitative performance differences were marginal; future studies based on large cohorts are warranted to investigate this aspect. Finally, we acknowledge that our study primarily included cases with unchanged or minimally progressed brain metastasis. This limitation may affect the generalizability of our findings, which will be addressed in a future work by expanding the dataset to include more diverse progression patterns to further validate and extend the proposed network.

In conclusion, a paired CycleGAN-based deep learning algorithm showed good performance in MRI harmonization, resulting in increased diagnostic confidence and potentially decreasing false positivity for the diagnosis of progression in FU MRI evaluation of brain metastasis patients. Patients with brain metastasis often undergo FU MR imaging using different scanners due to limited MRI resources. Our paired CycleGAN-based MRI harmonization technique may increase the diagnostic confidence in FU MRI evaluation in brain metastasis patients in such a clinical setting.

## Methods

This retrospective study was approved by the institutional review board of Seoul National University Hospital, and the requirement for informed consent was waived due to its retrospective nature. The study protocol was performed in accordance with the Declaration of Helsinki.

### Patients

**Fig. 3:**
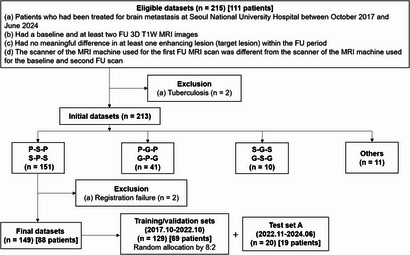
Flowchart for the study patient inclusion. P = Philips Healthcare, S = Siemens Healthineers, G = GE Healthcare. “P-S-P” refers to the dataset with the baseline imaging using a Philips scanner, the first follow-up (FU) imaging using a Siemens scanner, and the second FU imaging using a Philips scanner. “S-G-S” refers to the dataset with the baseline imaging using a Siemens scanner, the first FU imaging using a GE Healthcare scanner, and the second FU imaging using a Siemens scanner.

We searched our radiology report database and retrieved a total of 215 datasets from 111 consecutive patients who had been treated for brain metastasis between October 2017 and June 2024 (Fig. 3). The inclusion criteria were as follows: the patient (a) had been treated for brain metastasis at Seoul National University Hospital between October 2017 and June 2024; (b) had a baseline and at least two FU 3D T1W MRI images; (c) had no meaningful difference in at least one enhancing lesion (target lesion) within the FU period; and (d) the MRI scanner of the first FU MRI scan was different from that of the baseline and second FU scans. Two experienced neuroradiologists (S.H.C. and R.E.Y. with 21 and 13 years of experience in radiology, respectively) independently assessed the baseline and second FU MR images, acquired using the same MR scanner, to ensure no meaningful difference existed for the size and shape of the target lesion within the FU period. Two datasets were excluded from the initial pool because they were finally confirmed as tuberculosis. After categorizing the datasets by scanner combination, we selected the Ingenia 3.0T CX (Philips Healthcare)-Magnetom Skyra (Siemens Healthineers) combination, which had the largest number of datasets. Additionally, two datasets were excluded due to registration failure.

As a result, our study finally included 149 datasets from 88 patients with brain metastasis. The datasets between October 2017 and October 2022 (*n* = 129) from 69 patients were randomly divided into training and validation sets at an 8:2 ratio, resulting in 103 datasets for the training set and 26 datasets for the validation set. The datasets between November 2022 and June 2024 (*n* = 20) from 19 patients were allocated to test set A (Fig. 3). In addition, 17 datasets from 16 patients with brain metastases that showed noticeable size changes between November 2022 and June 2024 were allocated to test set B (change set) to validate the generalizability of the proposed model.

### MRI protocols

MRI was performed at a 3.0T imaging unit (Magnetom Skyra, Siemens Healthineers; Ingenia CX 3.0T, Philips Healthcare) with a 64-channel or a 32-channel head coil. The MRI protocol included 3D T1W magnetization-prepared rapid acquisition gradient echo sequence (MPRAGE) before and after the injection of gadobutrol (Gadovist; Bayer, Berlin, Germany; at a dose of 0.1 mmol/kg of body weight). Three consecutive scans were acquired; the first and third scans were obtained on the same MRI scanner from the same vendor using an identical scanning protocol, whereas the second scan was obtained on a different scanner from a different vendor using a different protocol. Specific imaging parameters for the 3D T1W MR sequence from all scanners are provided in Supplementary Table 4.

### Development of the deep learning algorithm

#### Data preprocessing

To prepare the MR images for model training and evaluations, several preprocessing steps were performed to ensure consistency and alignment across the dataset. First, the images were resampled to a 256 × 256 × 256 voxel grid and resampled to an isotropic voxel size of 1 × 1 × 1 mm³ using the FreeSurfer software package (version 7.2, http://surfer.nmr.mgh.harvard.edu/). To ensure consistent evaluation based on pixel intensity differences and maintain the stability during training process, the intensity values of each image were linearly scaled to a range of 0–1 using 32-bit precision.

**Fig. 4:**
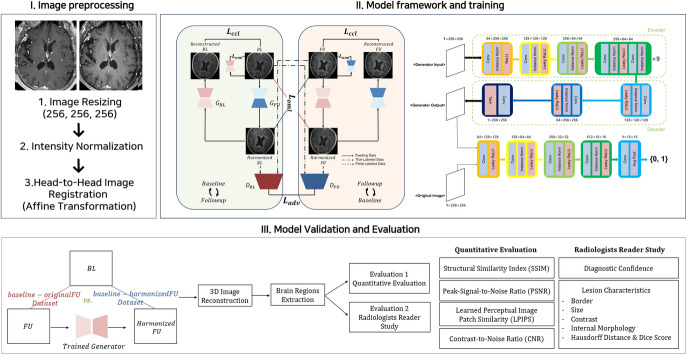
The workflow diagram of the proposed paired CycleGAN-based deep learning algorithm with its model architecture. (**a**) Manual MRI image preprocessing was performed before the training, validation, and evaluation processes in three steps. (**b**) The detailed architectures of the proposed network, generators, and discriminators. The generators, denoted as ${G}_{BL}$and ${G}_{FU}$, are trained to map images to the baseline and follow-up (FU) target domains, respectively. The green baseline-to-FU and the orange FU-to-baseline harmonization processes occurred simultaneously in a single training iteration. The loss functions were averaged from the values obtained from both the green and orange processes. (**c**) The flow chart details the steps of the model validation and evaluation. Evaluation 1 represents the computational comparison of image similarity metrics and CNRs between the original (baseline-original FU) and harmonized (baseline-harmonized FU) datasets. Evaluation 2 was the reader study scored by two trained neuroradiologists.

For spatial alignment, FU images were registered to their corresponding baseline images using a rigid-body affine transformation implemented in SPM12 with default parameters, ensuring them to be aligned within the same coordinate space. This approach enables consistent longitudinal intensity comparison between baseline and FU images, which is the primary focus of this study. The images were then split into axial slices, and the upper and lower 10% were excluded to reduce potential artifacts and irrelevant regions. Finally, the processed images were provided to the model in pairs, facilitating the learning of the spatial correspondences between baseline and FU images effectively (Fig. 4). This preprocessing pipeline was applied identically to all images used in the study, including baseline, original FU, and harmonized FU images. All similarity metrics and CNR analyses were computed using these preprocessed images.

#### Network training with loss functions

The goal of the proposed network was to train the style of pairwise data, a baseline image and a FU image, while maintaining the spatial information in brain regions. The overall framework of the model is shown in Fig. 4. Our model consisted of two generators, G_A_ and G_B_, and two discriminators, D_A_ and D_B_. The generators transformed an input image x into an output image G_A_(x) or G_B_(x), that embodied the style of the baseline or FU domain. Then, the discriminators were trained to classify whether an image x was a real image or a harmonized image through binary classification. The expectation operator $E\left[\cdot \right]$ denotes averaging over training images, with ${\mathrm{E}}_{x}$ referring to images x from either the BL or FU domain.

*Adversarial loss* We applied the following adversarial loss to train the generator such that the discriminator could not distinguish between the original image and the generated image G(x).

$$Adversarial\;Loss\left( {L_{{adv}} } \right) = E_{x} \left[ {\log D\left( x \right)} \right] + E_{x} \left[ {\log (1 - D(G\left( x \right))} \right]$$

The generators took an image x as inputs and learned to generate an output image G(x) that was evaluated by the discriminators. Building on the adversarial loss, we added additional loss functions to enforce specific conditions.

*Cycle-consistency loss* An additional cycle consistency loss^18^ was defined as the difference in pixel values between original images and reconstructed images ${G}_{B}\left({G}_{A}\left({x}_{B}\right)\right)$ as follows:

$$Cycle\;Consistency\;Loss\left( {L_{{ccl}} } \right) = E_{x} \left[ {\left\| {x_{B} - G_{B} \left( {G_{A} \left( {x_{B} } \right)} \right)} \right\|_{1} } \right]$$

where A and B refer to one of the two domains, either the baseline domain or FU domain, and x refers to one of the two original images, X and Y. Through the cycle consistency loss, the original structures of the brain regions could be preserved while changing the style of the domain during the training process. The baseline and FU domains were computed sequentially within a single training iteration and then aggregated into the final loss.

*Original Matching Loss* In the process of head-to-head style harmonization, the most crucial aspect is ensuring that the generator preserves the structural coherence of the original images. Therefore, we included the newly defined loss function which was developed based on the identity mapping loss^20^ and the L1 reconstruction loss used in Pix2Pix^21^, imposing an integrated penalty on each generator to learn as closely as possible to the original images, minimizing the intensity difference between x and generated images.

$$\begin{aligned} Original\;Matching\;Loss\left( {L_{{oml}} } \right) = & E_{x} \left[ {\left\| {x_{A} - G_{A} \left( {x_{B} } \right)} \right\|_{1} } \right] + E_{x} \left[ {\left\| {x_{A} - G_{A} \left( {x_{A} } \right)} \right\|_{1} } \right] \\ + E_{x} \left[ {\left\| {x_{B} - G_{B} \left( {x_{A} } \right)} \right\|_{1} } \right] + E_{x} \left[ {\left\| {x_{B} - G_{B} \left( {x_{B} } \right)} \right\|_{1} } \right] \\ \end{aligned}$$

This loss function helped to maintain the spatial information of the original images, preventing the alteration of clinically important structural regions, where A and B denote the BL or FU domain, and x represents the original input image. While the identity mapping loss in CycleGAN applies only to inputs already in the target domain and the Pix2Pix L1 loss enforces paired image fidelity^18,21^, our original matching loss enforces structural consistency across all generator–domain combinations, with equal weighting across terms, making it particularly appropriate for longitudinal harmonization where within-subject anatomical preservation is critical.

Based on the loss functions, we defined our final objective function as follows:

$$Final\;Loss\left( {L_{{final}} } \right) = \lambda _{{adv}} \cdot L_{{adv}} + \lambda _{{ccl}} \cdot L_{{ccl}} + \lambda _{{oml}} \cdot L_{{oml}}$$

Since we integrated the losses that regulated different structural and clinical contents, each loss was balanced by hyperparameter values that controlled the strength of regularization. Especially, the model needed to transfer the style from the source image while preserving the style-independent anatomical structures. We utilized the Adam optimizer with the β_1_ set to 0.5 and the β_2_ set to 0.99^22^. The learning rate for both the generator and discriminator was set to 2 × 10^−4^. To achieve stable convergence of the objective function, the learning rate was linearly reduced to 0 over 250,000 iterations during 50 epochs. The model was trained and tested on a NVIDIA RTX 3090 with 24 GB of memory and a batch size of 16. The whole training process took approximately 0.44 min per patient, with a total training time of approximately 16.67 h. The generation of inference images for evaluation, which included 3D image reconstruction, took approximately 450 s per patient. During the inference, harmonized 2D slices were stacked along the axial axis to reconstruct 3D volumes, preserving the original spatial resolution and metadata. Image intensities were then restored to the original range by applying the inverse transformation of the normalization.

#### Image similarity metrics for model evaluations

We evaluated the training progress of the proposed model with two training metrics. The structural similarity index measure (SSIM) was employed to assess the similarity between the baseline and harmonized FU images, considering aspects such as brightness, contrast, and structure^23^.

$$SSIM\left( {x,y} \right) = \frac{{\left( {2\mu _{x} \mu _{y} + c_{1} } \right)(2\sigma _{{xy}} + c_{2} )}}{{\left( {\mu _{x}^{2} + \mu _{y}^{2} + c_{1} } \right)(\sigma _{x}^{2} + \sigma _{y}^{2} + c_{2} )}}$$

SSIM compares the mean (µ), variance (σ^2^), and covariance (σ_xy_) of two images. Stabilization constants c_1_ and c_2_ prevent numerical instability, ensuring robust comparison. This metric integrates information on brightness, contrast, and structural similarity into a single measure and is commonly used for image similarity assessment. A higher SSIM score signifies a stronger resemblance between two images.

Additionally, we used the learned percentual image patch similarity (LPIPS) loss to gauge the perceptual similarity between the baseline and harmonized FU images based on a deep neural network trained to predict human perceptual judgments^24^.

$$LPIPS(x,y) = \sum\limits_{l} {\frac{1}{{H_{l} W_{l} }}} \sum\limits_{{h,w}} {\left\| {\phi _{l} \left( x \right)_{{h,w}} - \phi _{l} \left( y \right)_{{h,w}} } \right\|_{2}^{2} }$$

where ϕ_l_ represents the feature maps of images x and y extracted from the layer $l$ of a pre-trained model. H_l_ and W_l_ denote the height and width of the feature map at layer $l$ with the squared Euclidean distance between corresponding features. A lower LPIPS loss indicates greater similarity between the baseline and harmonized FU images.

Lastly, we employed the peak-signal-to-noise ratio (PSNR) to evaluate the reconstruction quality of the harmonized images. PSNR evaluates the ratio of the signal’s maximum power to the noise that affects its fidelity to assess the reconstruction quality and degree of image distortion (Jayant, N. S., & Noll, P., 1984).

$$PSNR(x,y)=10 \cdot {\mathrm{log}}_{10}\frac{L}{MSE(x,y)}$$

where x and y are the baseline and harmonized images, L is the maximum possible pixel intensity value, and MSE(x, y) is the mean squared error between the images with the total number of pixels. A higher PSNR value indicates better image quality with less distortion in the processed images.

#### Ablation study for model optimization

In this study, we introduced an original matching loss designed to maintain structural coherence and reduce distortions that may arise during harmonization. Given that preserving anatomical detail is critical in head-to-head harmonization, we conducted ablation experiments by adjusting the coefficient of the original matching loss ${\lambda}_{oml}$ (1,5,10 and 15). We assessed the results using three similarity metrics—SSIM, PSNR and LPIPS—which are described in detail in 2.3.3. Based on the performance across these metrics in the validation set, we identified the optimal ${\lambda}_{oml}$ and model checkpoints that achieve the best balance between structural preservation and overall image quality.

#### Comparison with other harmonization methods in test sets

The proposed model was compared against one conventional normalization technique and several deep learning–based style transfer approaches: intensity histogram matching, Pix2Pix^21^, STGAN^25^, and original CycleGAN (with and without identity loss). Intensity histogram matching is a non–deep learning method that standardizes image intensity distributions by mapping them to a reference distribution. In contrast, deep-learning based approaches, including the supervised Pix2Pix, unsupervised STGAN, and CycleGAN variants, learn cross-domain mappings through training. To ensure a fair and meaningful evaluation, all deep learning models were retrained on our dataset rather than using pretrained weights. Pix2Pix was included as a supervised benchmark to evaluate structural preservation since paired data are available. Especially, in case of STGAN, retraining was essential as our experiments were conducted on T1ce images—rather than standard T1-weighted images—and excluded skull stripping and MNI space registration, which differ from the default settings of the original implementations. For the CycleGAN-based comparisons, the original CycleGAN without the identity loss evaluated the effect of the proposed original matching loss under equivalent conditions and ensured controlled comparison between the original and the proposed model. The original CycleGAN with the identity loss was also included to rigorously evaluate our proposed Original Matching Loss against existing structural-preservation constraints.

Except for the necessary adaptations to our dataset, all other experimental settings, including network architectures and hyperparameters, strictly followed the respective original implementations of each method. Detailed experimental settings and implementation details for all comparison methods are provided in the Supplementary Methods.

During the evaluations in the unseen test sets, several quantitative evaluation metrics were used to assess the structural and anatomical preservation of the generated images by the trained model. As a preparation step, a volume-based parcellation using FreeSurfer software package Version 7.2 (http://surfer.nmr.mgh.harvard.edu/) was performed to obtain masks for brain subregions^26^. For quantitative assessment, we first calculated SSIM and PSNR scores between baseline and original FU images, as well as between baseline and harmonized FU images. Furthermore, contrast-to-noise ratios (CNRs) of the various brain regions were also calculated as follows (Patterson and Foster, 1983; Rodriguez-Molares et al., 2018):

$$CNR = \frac{{Mean\;signal\;intensity\left( {ROI} \right) - Mean\;signal\;intensity\left( {background} \right)}}{{Standard\;deviation\left( {background} \right)}}$$

### Reader study for the evaluation of the clinical utility of the model

Two experienced neuroradiologists (S.W.C. and Y.H.J. with 14 and 7 years of expertise, respectively) independently assessed the presence or absence of changes in the lesion characteristics on the first FU images (the original FU and the harmonized FU images) and their diagnostic confidence in the judgment. Each reader examined each patient’s MRI twice, resulting in a total of 258 brain MRIs assessed during two distinct review sessions, with a minimum two-week interval between them. In each session, the readers were presented with 129 datasets comprising a combination of baseline−original FU datasets and baseline−harmonized FU datasets, which were randomized and presented in a crossover fashion. The presence or absence of changes in lesion characteristics was analyzed in terms of the border, size, contrast enhancement, and internal morphology (e.g., size of the necrotic or cystic cavity) of the contrast-enhancing lesions. The diagnostic confidence was evaluated using a five-point scale as follows: 1 = none, 0–4%; 2 = poor, 5–35%; 3 = moderate, 36–65%; 4 = high, 66–95%; and 5 = excellent, 96–100%. In cases with multiple lesions, the largest lesion was selected as the target lesion for evaluation and only one representative lesion was evaluated per image. The readers were blinded to the information that the dataset only comprised cases with no changes in the FU to avoid any potential bias. In addition, the two readers segmented the target lesions in test set A, using ITK-SNAP software (version 3.8.0, http://www.itksnap.org/pmwiki/pmwiki.php), to calculate the Dice scores and Hausdorff distances of the target lesions between the baseline and FU images. The presence or absence of changes in lesion characteristics was also evaluated by the readers in the same manner for test set B (change set).

### Statistical analysis

Statistical software (MedCalc, version 11.1.1.0, Mariakerke, Belgium) was used to perform all statistical analyses. The Kolmogorov-Smirnov test was used to check normality for each parameter. For non-normally distributed variables including image similarity metrics and regional CNR values of brain regions, paired comparisons between baseline and FU images were performed using the Wilcoxon signed-rank test, for both original and harmonized data. For variables that satisfied normality assumptions, such as diagnostic confidence, paired t-tests were applied. The incidence of cases read to be unchanged at FU was compared between the original and harmonized FU images using the McNemar test. The average Hausdorff distance and Dice scores of the target lesions between the baseline and original FU images were compared with those between the baseline and harmonized FU images, using the Wilcoxon signed-rank test. *P* values less than 0.05 were considered to indicate statistical significance in all tests.

## Supplementary Information

Below is the link to the electronic supplementary material.

Supplementary Material 1

## Data Availability

The datasets generated during and/or analysed during the current study are available from the corresponding authors on reasonable request.

## Code availability

The code for the proposed method is publicly available at https://github.com/Iceberg6618/CycleGAN-Harmonization. Detailed instructions, including installation, dataset preparation, and example usage, are provided in the repository’s README file. Users can directly access and run the code to reproduce the experiments and evaluate the method.

## References

[1] Kim, T. et al. Epidemiology of Intracranial Metastases in Korea: A National Cohort Investigation. *Cancer Res. Treat.* **50**, 164–174. 10.4143/crt.2017.072 (2018). 28324921 10.4143/crt.2017.072 PMC5784640

[2] Hatiboglu, M. A., Akdur, K. & Sawaya, R. Neurosurgical management of patients with brain metastasis. *Neurosurg. Rev.* **43**, 483–495. 10.1007/s10143-018-1013-6 (2020). 30058049 10.1007/s10143-018-1013-6

[3] Zuo, L. et al. Unsupervised MR harmonization by learning disentangled representations using information bottleneck theory. *Neuroimage* **243**, 118569. 10.1016/j.neuroimage.2021.118569 (2021). 34506916 10.1016/j.neuroimage.2021.118569 PMC10473284

[4] Stamoulou, E. et al. Harmonization Strategies in Multicenter MRI-Based Radiomics. *J. Imaging*. **8**10.3390/jimaging8110303 (2022). 10.3390/jimaging8110303 PMC9695920 36354876

[5] Hu, F. et al. Image harmonization: A review of statistical and deep learning methods for removing batch effects and evaluation metrics for effective harmonization. *Neuroimage* **274**, 120125. 10.1016/j.neuroimage.2023.120125 (2023). 37084926 10.1016/j.neuroimage.2023.120125 PMC10257347

[6] Abbasi, S. et al. Deep learning for the harmonization of structural MRI scans: a survey. *Biomed. Eng. Online*. **23**10.1186/s12938-024-01280-6 (2024). 10.1186/s12938-024-01280-6 PMC11365220 39217355

[7] Fu, X. Digital Image Art Style Transfer Algorithm Based on CycleGAN. *Comput. Intell. Neurosci.* **2022**(6075398). 10.1155/2022/6075398 (2022). 10.1155/2022/6075398 PMC8776490 35069722

[8] Kang, S. K. et al. Synthetic CT generation from weakly paired MR images using cycle-consistent GAN for MR-guided radiotherapy. *Biomed. Eng. Lett.* **11**, 263–271. 10.1007/s13534-021-00195-8 (2021). 34350052 10.1007/s13534-021-00195-8 PMC8316520

[9] Kalantar, R. et al. CT-Based Pelvic T(1)-Weighted MR Image Synthesis Using UNet, UNet + + and Cycle-Consistent Generative Adversarial Network (Cycle-GAN). *Front. Oncol.* **11**, 665807. 10.3389/fonc.2021.665807 (2021). 34395244 10.3389/fonc.2021.665807 PMC8363308

[10] Kawahara, D. & Nagata, Y. T1-weighted and T2-weighted MRI image synthesis with convolutional generative adversarial networks. *Rep. Pract. Oncol. Radiother*. **26**, 35–42. 10.5603/RPOR.a2021.0005 (2021). 33948300 10.5603/RPOR.a2021.0005 PMC8086713

[11] Gebre, R. K. et al. Cross-scanner harmonization methods for structural MRI may need further work: A comparison study. *Neuroimage* **269**, 119912. 10.1016/j.neuroimage.2023.119912 (2023). 36731814 10.1016/j.neuroimage.2023.119912 PMC10170652

[12] Dikici, E. et al. Automated Brain Metastases Detection Framework for T1-Weighted Contrast-Enhanced 3D MRI. *IEEE J. Biomed. Health Inf.* **24**, 2883–2893. 10.1109/JBHI.2020.2982103 (2020). 10.1109/JBHI.2020.2982103 32203040

[13] Kaufmann, T. J. et al. Consensus recommendations for a standardized brain tumor imaging protocol for clinical trials in brain metastases. *Neuro Oncol.* **22**, 757–772. 10.1093/neuonc/noaa030 (2020). 32048719 10.1093/neuonc/noaa030 PMC7283031

[14] Nyul, L. G., Udupa, J. K. & Zhang, X. New variants of a method of MRI scale standardization. *IEEE Trans. Med. Imaging*. **19**, 143–150. 10.1109/42.836373 (2000). 10784285 10.1109/42.836373

[15] Fortin, J. P. et al. Removing inter-subject technical variability in magnetic resonance imaging studies. *Neuroimage* **132**, 198–212. 10.1016/j.neuroimage.2016.02.036 (2016). 26923370 10.1016/j.neuroimage.2016.02.036 PMC5540379

[16] Tian, D. et al. A deep learning-based multisite neuroimage harmonization framework established with a traveling-subject dataset. *Neuroimage* **257**, 119297. 10.1016/j.neuroimage.2022.119297 (2022). 35568346 10.1016/j.neuroimage.2022.119297

[17] Zuo, L. et al. Information-based disentangled representation learning for unsupervised MR harmonization. in *Information Processing in Medical Imaging.* (eds Aasa Feragen et al.) 346–359 (Springer International Publishing).

[18] Zhu, J. Y., Park, T., Isola, P. & Efros, A. A. Unpaired image-to-image translation using cycle-consistent adversarial networks. in *Proceedings of the IEEE International Conference on Computer Vision* 2223–2232.

[19] Zhang, H., Li, H., Dillman, J. R., Parikh, N. A. & He, L. Multi-contrast MRI image synthesis using switchable cycle-consistent generative adversarial networks. *Diagnostics (Basel)*. 10.3390/diagnostics12040816 (2022). 10.3390/diagnostics12040816 PMC9026507 35453864

[20] Taigman, Y., Polyak, A. & Wolf, L. Unsupervised cross-domain image generation. in *ICLR* arXiv:1611.02200. (2017). https://openreview.net/forum?id=Sk2Im59ex

[21] Isola, P., Zhu, J. Y., Zhou, T. & Efros, A. A. Image-to-image translation with conditional adversarial networks. In *Proceedings of the IEEE Conference on Computer Vision and Pattern Recognition* 1125–1134 (2017).

[22] Kingma, D. P., Ba, J. & Adam A Method for Stochastic Optimization. arXiv:1412.6980 (2014). https://ui.adsabs.harvard.edu/abs/2014arXiv1412.6980K

[23] Wang, Z., Bovik, A. C., Sheikh, H. R. & Simoncelli, E. P. Image quality assessment: from error visibility to structural similarity. *IEEE Trans. Image Process.* **13**, 600–612. 10.1109/tip.2003.819861 (2004). 15376593 10.1109/tip.2003.819861

[24] Zhang, R., Isola, P., Efros, A. A., Shechtman, E. & Wang, O. The unreasonable effectiveness of deep features as a perceptual metric. *Proc. Cvpr Ieee* 586–595. 10.1109/Cvpr.2018.00068 (2018).

[25] Gao, Y., Liu, Y., Wang, Y., Shi, Z. & Yu, J. A. Universal intensity standardization method based on a many-to-one weak-paired cycle generative Adversarial network for magnetic resonance images. *IEEE Trans. Med. Imaging*. **38**, 2059–2069. 10.1109/TMI.2019.2894692 (2019). 30676951 10.1109/TMI.2019.2894692

[26] Fischl, B. FreeSurfer. *Neuroimage* **62**, 774–781, doi:10.1016/j.neuroimage.2012.01.021 (2012). 10.1016/j.neuroimage.2012.01.021 PMC3685476 22248573

