# Supplementary material for: Development of head-to-head and longitudinal CycleGAN algorithm for MRI harmonization: validation in follow-up MRI evaluation in patients with brain metastasis
Source: Sci Rep. 2026 Mar 11;16:13163. doi: 10.1038/s41598-026-43755-7 (PMC13103079; doi:10.1038/s41598-026-43755-7)
Supplement: Supplementary file 1 — Supplementary Material 1 [file 41598_2026_43755_MOESM1_ESM.docx]

**Supplementary Results**

*Reader Study for the Evaluation of Clinical Utility of the Model in Test Set B (Change Set)*

To test whether the harmonization algorithm also accounts for image changes resulting from the subject's disease status, we have conducted additional analyses for 17 datasets from 16 patients in whom brain metastases changed in size during the FU. The incidence of cases read to be ‘changed’ did not significantly differ between original FU images and harmonized FU images (Supplementary Table 3). The diagnostic confidence either increased (Reader 1: median, 4.0 [IQR, 3.0−4.0] vs. median, 5.0 [IQR, 5.0−5.0]; *P* <.001) or remained similar (Reader 2: median, 4.0 [IQR, 3.0−4.3] vs. median, 4.0 [IQR, 3.8−5.0]; *P* = .20) after the harmonization.

**Supplementary Methods**

*Implementation Details of Comparison Models*

This sections provides detailed descriptions of the implementation and experimental configurations of all comparison methods used in this study. To ensure a fair and reproducible evaluation, all comparisons were applied under an identical preprocessing pipeline as described in Section 4.3.1, and these steps were implemented consistently across histogram matching, STGAN, Pix2Pix, CycleGAN, and the proposed network.

Histogram matching was implemented as a conventional, non–learning-based intensity normalization baseline using the HistogramMatchingImageFilter (<https://simpleitk.org/doxygen/latest/html/classitk_1_1simple_1_1HistogramMatchingImageFilter.html>) from the SimpleITK library. The follow-up image was intensity-matched to the corresponding baseline image using 256 histogram levels and 10 match points, with mean intensity thresholding enabled to reduce the influence of extreme outlier voxels. These parameters follow commonly adopted default configurations in medical image processing and were selected to provide a conservative baseline approach without introducing additional spatial distortions. Importantly, histogram matching was applied only after all spatial alignment steps were completed, and no additional transformations beyond intensity remapping were performed.

For the Pix2Pix baseline, we implemented a conditional Generative Adversarial Network (cGAN) to evaluate the performance of supervised image-to-image translation. Specifically, the model was trained in two separate directions to account for vendor-specific characteristics: from SIEMENS to Philips and from Philips to SIEMENS.

For the STGAN baseline, the model was trained from scratch on our dataset rather than initialized with pretrained weights. This was necessary because our experimental pipeline differed from the original STGAN setting in terms of input modality and preprocessing, including the use of postcontrast T1W images and the exclusion of skull stripping and MNI space normalization. Aside from these modality- and preprocessing-related differences, the original STGAN architecture, loss formulation, and training protocol were preserved to ensure a controlled and fair comparison.

The original CycleGAN was trained using two primary loss components (adversarial loss and cycle-consistency loss) with or without the identity loss. The original CycleGAN without the identity loss evaluated the effect of the proposed original matching loss under equivalent conditions and ensured controlled comparison between the original and the proposed model. The original CycleGAN with the identity loss was also included to rigorously evaluate our proposed Original Matching Loss against existing structural-preservation constraints. The original CycleGANs were implemented under the same computational environment and training configuration as the proposed model. The weights of the adversarial loss and cycle-consistency loss were set identically as 1 and 5 for both the CycleGAN baseline and the proposed method. Additionally, all other training settings, including optimizer parameters (Adam β_1_ = 0.5, β_2_ = 0.99), learning rate (2×10^-4^), learning epochs (50), and computational resource (NVIDIA RTX 3090 with 24GB) were kept the same. This fixed model settings ensured that differences in performance between the CycleGAN baseline and the proposed approach were attributable solely to the inclusion of the original matching loss.

**Supplementary Table 1.** PSNR and SSIM scores between baseline and FU images in test set B (change set)

|  | | PSNR (vs. BL) | *P* Value | SSIM (vs. BL) | *P* Value |
| --- | --- | --- | --- | --- | --- |
| Original FU | | 21.59 ± 1.60 | - | 0.775 ± 0.067 | - |
| Histogram Matching | | 22.53 ± 1.58 | <.001 | 0.853 ± 0.035 | <.001 |
| Pix2Pix | | 22.75 ± 1.11 | <.01 | 0.855 ± 0.023 | <.001 |
| STGAN | | 22.95 ± 1.47 | .001 | **0.865 ± 0.010** | <.001 |
| Original CycleGAN (with Identity Loss) | | 22.98 ± 1.24 | <.01 | 0.861 ± 0.030 | <.001 |
| Original CycleGAN (without Identity Loss) | | 23.07 ± 1.37 | <.001 | 0.853 ± 0.041 | <.001 |
| CycleGAN (Ours) | **23.23 ± 1.34** | | <.001 | 0.857 ± 0.038 | <.001 |

**Note.**— Data are means ± standard deviations. CycleGAN (Ours) incorporates an additional original matching loss with a coefficient of 10 ($\lambda_{oml}=10)$, while all other parameters are consistent with the original CycleGAN without Identity Loss. Adversarial loss ($\lambda_{adv})$ and Cycle Consistency loss ($\lambda_{ccl})$ are fixed at 1 and 5, respectively. Each score was evaluated volume-wise, and LPIPS was excluded since it is originally designed for 2D image comparison and is not directly applicable to 3D volume data. For each harmonization method, similarity scores obtained from BL–harmonized FU pairs were statistically compared with those from BL–original FU pairs using the Wilcoxon signed-rank test. *P* values less than 0.05 were considered statistically significant.

BL = baseline; FU = follow-up; PSNR = peak signal-to-noise ratio; SSIM = structural similarity index measure.

**Supplementary Table 2.** Differences in the CNRs between baseline and FU images in test set B (change set)

| Regions |  |  | |  | | |  | |  | |  | | CNR Difference | | | | | | |
| --- | --- | --- | --- | --- | --- | --- | --- | --- | --- | --- | --- | --- | --- | --- | --- | --- | --- | --- | --- |
|  | Original FU | | Histogram Matching | | *P* Value | Pix2Pix | | *P* value | | STGAN | | *P* Value | | Original CycleGAN (with Identity Loss) | *P* Value | Original CycleGAN (without Identity Loss) | *P* Value | CycleGAN (Ours) | *P* Value |
| Amygdala | 0.645±0.232 | | 0.595±0.232 | | 0.10 | **0.180 ± 0.110** | | <.001 | | 0.250±0.106 | | <.001 | | 0.253 ± 0.139 | <.001 | 0.209±0.114 | <.001 | 0.230±0.133 | <.001 |
| Brainstem | 0.987±0.316 | | 1.050±0.341 | | <.05 | **0.250 ± 0.136** | | <.001 | | 0.400±0.167 | | <.001 | | 0.290 ± 0.117 | <.001 | 0.355±0.154 | <.001 | 0.321±0.159 | <.001 |
| Caudate | 0.604±0.258 | | 0.564±0.210 | | 0.05 | 0.194 ± 0.148 | | <.001 | | **0.148±0.068** | | <.001 | | 0.277 ± 0.155 | <.001 | 0.217±0.117 | <.001 | 0.224±0.144 | <.001 |
| Cerebellum WM | 0.971±0.323 | | 1.010±0.396 | | 0.24 | **0.245 ± 0.133** | | <.001 | | 0.398±0.188 | | <.001 | | 0.265 ± 0.139 | <.001 | 0.314±0.171 | <.001 | 0.286±0.163 | <.001 |
| Cerebellum GM | 0.390±0.198 | | 0.373±0.163 | | 0.50 | 0.163 ± 0.113 | | <.001 | | 0.140±0.097 | | <.001 | | 0.156 ± 0.097 | <.001 | 0.135±0.094 | <.001 | **0.134±0.090** | <.001 |
| Cerebral WM | 0.670±0.267 | | 0.597±0.205 | | <0.01 | **0.146 ± 0.116** | | <.001 | | 0.189±0.103 | | <.001 | | 0.220 ± 0.145 | <.001 | 0.205±0.113 | <.001 | 0.221±0.169 | <.001 |
| Insula Ctx | 0.380±0.218 | | 0.380±0.148 | | 1.00 | 0.176 ± 0.117 | | .001 | | **0.094±0.066** | | <.001 | | 0.198 ± 0.131 | <.001 | 0.147±0.092 | <.001 | 0.183±0.120 | <.01 |
| Pallidum | 0.948±0.311 | | 1.019±0.392 | | 0.10 | **0.215 ± 0.144** | | <.001 | | 0.278±0.182 | | <.001 | | 0.309 ± 0.170 | <.001 | 0.279±0.179 | <.001 | 0.293±0.175 | <.01 |
| Putamen | 0.750±0.267 | | 0.697±0.268 | | <0.01 | **0.202 ± 0.125** | | <.001 | | 0.236±0.149 | | <.001 | | 0.295 ± 0.153 | <.001 | 0.235±0.129 | <.001 | 0.256±0.158 | <.001 |
| Thalamus Proper | 0.780±0.274 | | 0.853±0.287 | | <0.05 | **0.195 ± 0.130** | | <.001 | | 0.223±0.107 | | <.001 | | 0.250 ± 0.161 | <.001 | 0.212±0.151 | <.001 | 0.244±0.182 | <.001 |

**Note.**—Data are means ± standard deviations. The smallest CNR difference for each region is indicated in bold. *P* values were calculated using paired t-tests to assess statistical significance compared to the original FU images. Paired t-tests were used for statistical comparison, as the variables satisfied normality assumptions, to assess statistical significance compared to the original FU images.

BL = baseline; CNR = contrast-to-noise ratio; Ctx = cortex; FU = follow-up; GM = gray matter; WM = white matter.

**Supplementary Table 3.** Reader study for lesion characterization in test set B (change set)

|  | Original FU (n = 17) | Harmonized FU (n = 17) | *P* Value |
| --- | --- | --- | --- |
| Reader 1 | | | |
| Border | 8 (47) | 9 (53) | >.99 |
| Size | 17 (100) | 17 (100) | NA |
| Contrast enhancement | 10 (59) | 12 (71) | .62 |
| Internal morphology | 14 (82) | 13 (77) | >.99 |
| Reader 2 | | | |
| Border | 11 (65) | 11 (65) | >.99 |
| Size | 17 (100) | 17 (100) | NA |
| Contrast enhancement | 8 (47) | 10 (59) | .63 |
| Internal morphology | 15 (88) | 13 (77) | .50 |

Note.—Unless otherwise indicated, data represent numbers of cases interpreted to be ‘changed’ on the FU images (percentages). *P* values were calculated using the McNemar test to compare original and harmonized FU images for each reader.

**Supplementary Table 4.** MRI scan parameters

|  | Magnetom Skyra  (3D T1-weighted MPRAGE) | Ingenia 3.0T CX  (3D T1-weighted TFE) |
| --- | --- | --- |
| Field strength (T) | 3.0 | 3.0 |
| Head coil channel | 64 | 32 |
| TR (ms) | 1740 | 8.5 |
| TE (ms) | 3.0 | 4.6 |
| FA (°) | 12 | 8 |
| NEX | 1 | 1 |
| Matrix | 256×232 | 252×250 |
| Section thickness (mm) | 1 | 1 |
| Intersection gap (mm) | 0 | 0 |
| FOV (mm) | 227×250 | 220×220 |

FA, flip angle; FOV, field of view; MPRAGE, magnetization-prepared rapid gradient echo; NEX, number of excitations; TE, echo time; TFE, turbo-field imaging; TR, repetition time.
